# Cost-effectiveness analysis of oral fentanyl formulations for breakthrough cancer pain treatment

**DOI:** 10.1371/journal.pone.0179523

**Published:** 2017-06-27

**Authors:** Paolo Angelo Cortesi, Lucia Sara D’Angiolella, Renato Vellucci, Massimo Allegri, Giuseppe Casale, Carlo Favaretti, Flavia Kheiraoui, Giancarlo Cesana, Lorenzo Giovanni Mantovani

**Affiliations:** 1 Research Centre on Public Health (CESP), University of Milan-Bicocca, Monza, Italy; 2 Palliative Care and Pain Therapy Unit, University Hospital of Careggi, Florence, Italy; 3 Department of Surgical Sciences, Anesthesia Intensive Care and Pain Therapy Service, Azienda Ospedaliera, University of Parma, Parma, Italy; 4 ANTEA Center, Antea Formad Educational, Rome, Italy; 5 Institute of Public Health, Catholic University of the Sacred Heart, Rome, Italy; Jagiellonian University, POLAND

## Abstract

Breakthrough cancer Pain (BTcP) has a high prevalence in cancer population. Patients with BTcP reported relevant health care costs and poor quality of life. The study assessed the cost-effectiveness of the available Oral Fentanyl Formulations (OFFs) for BTcP in Italy. A decision-analytical model was developed to estimate costs and benefits associated with treatments, from the Italian NHS perspective. Expected reductions in pain intensity per BTcP episodes were translated into, percentage of BTcP reduction, resource use and Quality-Adjusted-Life-Years (QALYs). Relative efficacy, resources used and unit costs data were derived from the literature and validated by clinical experts. Probabilistic and deterministic sensitivity analyses were performed. At base-case analysis, Sublingual Fentanyl Citrate (FCSL) compared to other oral formulations reported a lower patient’s cost (€1,960.8) and a higher efficacy (18.7% of BTcP avoided and 0.0507 QALYs gained). The sensitivity analyses confirmed the main results in all tested scenarios, with the highest impact reported by BTcP duration and health care resources consumption parameters. Between OFFs, FCSL is the cost-effective option due to faster reduction of pain intensity. However, new research is needed to better understand the economic and epidemiologic impact of BTcP, and to collect more robust data on economic and quality of life impact of the different fentanyl formulations.

Different fentanyl formulations are available to manage BTcP in cancer population. The study is the first that assesses the different impact in terms of cost and effectiveness of OFFs, providing new information to better allocate the resources available to treat BTcP and highlighting the need of better data.

## Introduction

Pain continues to be an important issue in patients with cancer. About 60–90% of cancer patients die without be treated for pain [1]. Breakthrough cancer Pain (BTcP) is a transitory exacerbation of pain that occurs on a background of otherwise controlled, persistent pain [2]. BTcP has a rapid onset and a short duration time, and drugs used for chronic pain are generally not effective as short-acting treatments [3]. In studies of patients with chronic cancer pain, 30–90% of patients with controlled, persistent pain reported also BTcP [4–6]. A recent pooled analysis reported a BTcP prevalence of 59.2%, highlighting the high burden and frequency of this problem [7].

Inadequately treated, uncontrolled or poorly controlled pain is likely to cause consuming of more health care resources (e.g. pain-related hospitalizations and emergency department visits) and increasing treatment costs [2,4,8–12]. In addition to the high impact on health care costs, BTcP has a negative impact on quality of life (including activities of daily living, sleep, social relationships, and mood) and medical outcomes [13,14]. The significant economic impact of unrelieved cancer pain and its reduction with efficient pain management strategies has been well established [13–15]; however, few studies have assessed the costs related to BTcP and its treatment as well as indirect costs related to an appropriate/inappropriate management [13,14,16–19].

Oral opioids are often recommended for the management of BTcP, but usually the time for clinically relevant analgesia (30–60 minutes) may be longer than the time of peak intensity of many BTcP episodes [20–23]. Due to such a lag between the effect of drug and onset duration of BTcP, immediate release fentanyl formulations have been proposed as a better treatment option, based on a pharmacokinetics that mirrors the rapid onset of BTcP and on better clinical outcomes compared to oral morphine [22,24–26].

Currently, several fentanyl formulations are available with different pharmacodynamics profiles and way of administrations [27]. All these formulations are an effective treatment for BTcP, lowering pain intensity and offering higher relief compared to Placebo and oral morphine [24]. Despite the evidences about the efficacy of fentanyl, there are no cost-effectiveness analyses that could help clinicians and health decision makers in the selection of the best formulation. Aim of this study was to assess the cost-effectiveness of the different Oral Fentanyl Formulations (OFFs) for the treatment of BTcP, from Italian National Health System (NHS) perspective.

## Methods

A decision analytical model was developed to estimate the cost-effectiveness of OFFs for the treatment of BTcP in advance cancer stage patients, using Microsoft Excel and the Italian NHS perspective. According with short life expectancy of patients, a decision tree model was used to compare the treatment paths and resource use attributable to five fentanyl formulations: Sublingual Fentanyl Citrate (FCSL), Fentanyl Buccal Soluble Film (FBSF), Fentanyl Buccal Tablet (FBT), Oral Transmucosal Fentanyl Citrate (OTFC) and Fentanyl Sublingual Tablets (FST) (S1 Fig). The effects of these treatments, estimated as expected reductions in Pain Intensity (PI) of BTcP episodes, were assessed by the model and translated into cost differences and gains in Quality-Adjusted Life Years (QALYs). Forty-five days was assumed as time horizon for the simulation because it reflected the average treatment duration of cancer patients with rapid onset opioid in Italy, based on the opinion of clinical experts involved in the study. The model focused on a cohort of patients with advanced cancer stage who have begun a palliative care with a short life expectancy. No extended effect after interruption of treatment was simulated in the model. Effects and costs were not discontinued because the time horizon was lower than 1 year

### Population

The population simulated in the model was assumed to be similar to the people involved in the fentanyl clinical trials [28,29]: adult patients with advanced cancer stage (≥ 18 years), suffering from BTcP, experiencing one to four BTcP episodes per day and receiving stable opioid medication to control background pain.

The model base-case analysis assumed a background Pain Index (PI)of 2 on a Numerical Rating Scale (NRS), a frequency of 3 BTcP episodes per day and an average BTcP duration equal to 30 minutes (Table 1) [7, 26, 27, 29, 30].

**Table 1:** Model parameters: Base case.

Parameter	Value (UI)	References
Time horizon (days)	45.00 (30.00–60.00)	Assumption
BTcP duration (min)	30.00 (15.00–60.00)	[27]
BTcP episode per day	3.00 (2.00–3.50)	[7,27]
Background pain intensity (NRS)	2.00 (1.50–3.00)	[31]

UI = Uncertainty Intervals; BTcP = Breakthrough cancer Pain; NRS = Numeric Rating Scale.

### Treatments efficacy

**Fig 1:**
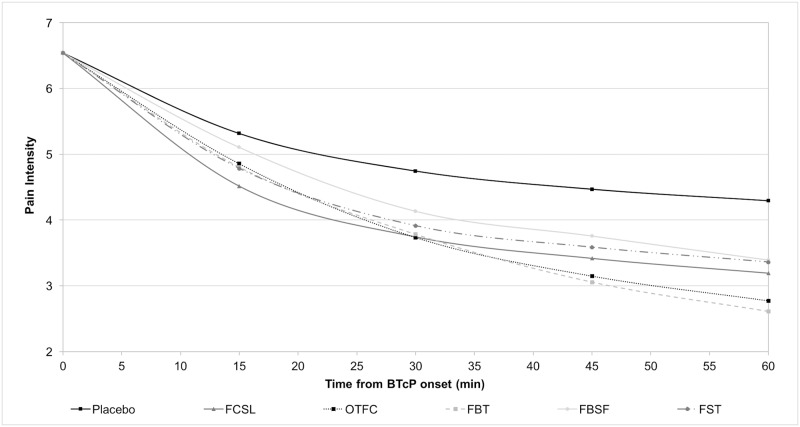
PI curves during a BTcP, derived from trials data. FCSL = Sublingual Fentanyl Citrate; OTFC = Oral Transmucosal Fentanyl Citrate; FBT = Fentanyl Buccal Tablet; FBSF = Fentanyl Buccal Soluble Film; FST = Fentanyl Sublingual Tablets.

The efficacy of different OFFs was assessed based on clinical trials and a meta-analysis of fentanyl formulations for the treatment of BTcP [28,29]. The effects of OFFs were reported in the model as Pain Intensity Difference (PID) at 15, 30, 45 and 60 minutes after administration for each treatment compared to Placebo (Table 2); if a time point for a given study was missing, an average was calculated using adjacent time points as proxy [17,28,29]. As already performed in the study of Vissers et al. [17], using the PI reported in the Placebo arms and the PID produced by each formulation, we created a series of PI curves (Fig 1). Based on this approach we estimated the total Area Under the Curve (AUC) at 15, 30, 45 and 60 minutes for each formulation [17]. AUC was calculated using a cumulative triangulation calculation [32] with data frequency of 15, 30, 45 and 60 minutes.

**Table 2:** Treatment efficacy and health care resources consumed by each fentanyl formulation.

Parameter	FST	FBSF	FBT	OTFC	FCSL	References
**Efficacy on BTcP event: PID Data for Individual formulations vs Placebo**						
0 min	-	-	-	-	-	[28, 29]
15 min	0.53	0.21	0.51	0.46	0.80	
30 min	0.83	0.61	0.96	1.01	1.00	
45 min	0.88	0.71	1.41	1.32	1.05	
60 min	0.93	0.90	1.68	1.52	1.10	
**Health care resources consumed (in 45 days)**						
GP visits, N	2.18	4.18	2.98	2.98	1.54	[19]
Specialist visits, N	1.13	2.17	1.55	1.55	0.80	
Hospitalizations, N	0.19	0.37	0.26	0.26	0.14	
Access to ER, N	0.44	0.84	0.60	0.60	0.31	
Physiotherapy, N	0.13	0.25	0.18	0.18	0.09	
Psycotherapy, N	0.85	1.63	1.16	1.16	0.60	
Acupuncture, N	0.08	0.14	0.10	0.10	0.05	
Transcutaneous electrical nerve stimulation, N	0.01	0.03	0.01	0.03	0.01	

FST = Fentanyl Sublingual Tablets; FBSF = Fentanyl Buccal Soluble Film; FBT = Fentanyl Buccal Tablet; OTFC = Oral Transmucosal Fentanyl Citrate; FCSL = Sublingual Fentanyl Citrate; BTcP = Breakthrough Cancer Pain; PID = Pain Intensity Difference; GP = General Practioner; ER = Emergency Room; N = Number.

The percentage reduction of AUC between each fentanyl formulation and Placebo was reported as percentage of BTcP (%BTcP) avoided to estimate the different efficacy of each fentanyl formulation [17]. Based on the specific number of BTcP episodes per day, the %BTcP avoided by each treatment was calculated for a time horizon of 45 days [17,27].

### Utilities

Utilities were incorporated in the model and associated to PI curves in order to estimate QALY gained for each OFFs. In the absence of specific utility values for BTcP in Italy, we used the estimated utilities by Vissers et al. [17]. In the Vissers et al. study, utility values were estimated using a Time Trade Off (TTO) approach [33]. In the TTO, 99 members of the United Kingdom general population assessed eight PI profiles, generated to represent possible different courses of PI over 60 minutes BTcP episodes. Because the eight PI profiles could not represent all possible courses of PI, a mathematical function was used to derive a relationship between utility and each possible AUC in the model [17]. With this data, Vissers et al. estimated the utility associated to the simulated treatment and the %BTcP avoided [17].

Based on these results, we estimated the relationship between utility and %BTcP avoided to assess the corresponding utility values calculated at 15, 30, 45 and 60 minutes. The utility was estimated with the following formula:

$$Utility=\left(\left(0.1765*e^{\left(0.0073*\%BTcP\right)}\right)*1.0082\right)*2$$

The details of the methods used to estimate this formula are reported in the data in S1 File.

### Costs

Under the NHS perspective, direct medical costs, namely those related to the treatment and management of BTcP (visits, access to Emergency Room (ER), hospitalizations and non-drug treatment, including physical therapy, psychotherapy, acupuncture and transcutaneous electrical nerve stimulation) were included in the model (Table 3). Costs were expressed in Euros (€, 2016).

**Table 3:** Model parameters: Costs.

Parameter	Value, €	References
OTFC single dose	€ 9.49	[35]
FST single dose	€ 8.51	[35]
FBT single dose	€ 9.68	[35]
FBSF single dose	€ 7.47	[35]
FCSL single dose	€ 9.68	[35]
GP visits	€ 12.90	[19]
Specialist visits	€ 20.70	[19]
Hospitalizations	€ 3,594.9	[19]
Access to ER	€ 384.80	[19]
Physiotherapy	€ 10.20	[19]
Psycotherapy	€ 19.40	[19]
Acupuncture	€ 8.50	[19]
Transcutaneous electrical nerve stimulation	€ 2.60	[19]

OTFC = Oral Transmucosal Fentanyl Citrate; FST = Fentanyl Sublingual Tablets; FBT = Fentanyl Buccal Tablet; FBSF = Fentanyl Buccal Soluble Film; FCSL = Sublingual Fentanyl Citrate; GP = General Practioner; ER = Emergency Room.

Existing tariffs were employed for the visits and hospitalizations [34]. The specific health care resources used and the associated costs for each formulation were retrieved from a recent budget impact analysis [19]. The health care resources consumption was estimated based on the average health care resources consumed by patients treated with fentanyl formulation reported in the Budget Impact Analysis (BIA), and based on a linear relationship between health care consumption and time to analgesic onset reported for each formulation [18,25,28]. The Public Price (PP) was considered to quantify the cost of drugs used [35] and was reported in Table 3.

The overall cost of the subjects with BTcP, treated with each formulation of fentanyl, was estimated considering the drug treatment cost and the other health resources consumed, assuming 3 BTcP episodes per day and a single administration of fentanyl per BTcP, for a time horizon of 45 days. The same adherence was assumed for all formulations and set to 100%. The adverse events and their related costs were not included in the model because of their reported low economic impact [18]. Consistent with the adopted perspective, non-medical direct and indirect costs were not included in the analyses.

### Analysis

The model results were expressed as %BTcP avoided, QALYs gained, incremental costs and QALYs, comparing the most effective formulation vs all the others. Costs and effects were combined to calculate the Incremental Cost Effectiveness Ratio (ICER) expressed as € per BTcP avoided and € per QALY gained. The base-case analysis was performed using the scenario with an average BTcP duration of 30 minutes, as reported in a recent survey conduct in Italy [27]. In the base-case analysis the %BTcP avoided was estimated considering the efficacy of the different formulations in the first 30 minutes from the BTcP onset. In the analyses a treatment was considered cost-effective if the ICER was lower than 50.000–60.000 € per QALY gained, the threshold value frequently used in Italy [36,37].

To confirm the robustness of the model assumption and the impact of each variable on the results, probabilistic and deterministic sensitivity analyses were performed. The deterministic analysis was performed to produce 6 alternative scenarios. In 3 of these scenarios, the treatment efficacy was changed assuming a BTcP duration of 15 (alternative scenario 1), 45 (alternative scenario 2) and 60 (alternative scenario 3) minutes. Further, because the used medical resources in the model were estimated based on time to treatment efficacy onset, an alternative approach was tested in the alternative scenario 4. This scenario assumed a linear relationship between resource consumption and %BTcP avoided (S1 Table), as performed in Vissers et al study [17]. Finally, the last deterministic analyses were performed changing the time horizons and assuming treatment duration of 30 and 60 days (alternative scenario 5 and 6). Performing different scenario analyses and testing a maximum and minimum value compared to base-case, the value ranges for deterministic sensitivity analysis were not reported.

Probabilistic sensitivity analysis was performed to assess the variability of all parameters considered in the model: 1,000 simulations were generated for the base-case scenario (S2 Table). The results of the probabilistic sensitivity analysis were reported by the Cost-effectiveness Acceptability Curve (CEAC).

## Results

The results of the base case simulations are reported in Table 4. The analyses showed a lower mean cost, a higher %BTcP reduction and QALY gained for FCSL compared to the other fentanyl formulations, at 30 minutes after-administration.

**Table 4:** Base-case analysis results.

Treatment	Costs, €	%BTcP avoided (vs Placebo)	QALYs (vs Placebo)	Incremental costs, €^	Incremental % BTcPavoided^	Incremental QALYs*	ICER (€ per BTcP avoided)	ICER (€ Cost per QALY gained)
**FCSL**	€1,960.76	-18.69%	0.0507	-	-	-	-	-
**FST**	€2,069.18	-13.59%	0.0489	-€ 108.43	-5.10%	0.0018	Dominated by FCSL	Dominated by FCSL
**FBSF**	€2,776.06	-7.40%	0.0468	-€ 815.31	-11.29%	0.0039	Dominated by FCSL	Dominated by FCSL
**FBT**	€2,565.94	-14.23%	0.0493	-€ 605.18	-4.46%	0.0015	Dominated by FCSL	Dominated by FCSL
**OTFC**	€2,540.36	-13.87%	0.0489	-€ 579.60	-4.82%	0.0018	Dominated by FCSL	Dominated by FCSL

BTcP = Breakthrough cancer Pain; QALYs = Quality Adjusted Life Years; ICER = Incremental Cost-Effectiveness Ratio; FCSL = Sublingual Fentanyl Citrate; FST = Fentanyl Sublingual Tablets FBSF = Fentanyl Buccal Soluble Film; FBT = Fentanyl Buccal Tablet; OTFC = Oral Transmucosal Fentanyl Citrate;

^ Negative value of incremental costs and %BTcP avoided means lower cost and higher %BTcP avoided with FCSL

*Positive value of incremental QALYs mean higher QALYs gained with FCSL.

In the base-case analysis, FCSL was more effective than other oral fentanyl formulations with an incremental percentage of %BTcP avoided of 4.5% vs FBT, 4.8% vs OTFC, 5.1% vs FST and 11.3% vs FBSF. The %BTcP avoided using FCSL was associated to an incremental QALY gained between 0.0015 vs FBT and 0.0039 vs FBSF in the simulated treatment period of 45 days (Table 4).

The analysis also reported FCSL treatment as the cheapest option, with an achievable cost saving for each patient that goes from a minimum of €108.4 vs FST to a maximum of €815.3 vs FBSF, respectively (Table 4). The cost-effectiveness analysis suggests that FCSL is the dominant treatment strategy for managing BTcP (i.e. more effective and less costly), in comparison with other treatments (Table 4).

## Sensitivity analysis

### Deterministic sensitivity analysis

In the alternative scenarios 1, 2 and 3, we have tested the impact of BTcP duration on the model results. Using 15, 45 and 60 minutes as BTcP duration, FCSL provided the greatest improvement in terms of QALY and BTcP reduction, vs FST and FBSF (Table 5). Compared to FBT and OTFC, FCSL showed a lower BTcP reduction and QALY improvement in the 45 and 60 minutes scenarios. FCSL was dominant compared to FBSF and FST in 15, 45 and 60 minutes scenarios and in the 15 and 45 minutes scenarios compared to FBT and OTFC. In the 60 minutes scenario, FBT and OTFC were not cost-effective compared to FCSL because they reported an ICER higher than the threshold value of 50.000–60.000 € per QALY. Table 5 showed the relative effects of various changes to health resource consumption, used in the model (alternative scenario 4). In this scenario, FCSL was slightly more expensive compare to FST and FBSF but it remained cost-effectiveness with an ICER per QALY gained of €27,661 vs FST and €15,355 vs FBSF. FCSL was still dominant when compared to FST and FBSF.

**Table 5:** Deterministic sensitivity analysis: Results of the 7 alternative scenarios tested.

FCSL vs	Incremental costs, €	Incremental %BTcP avoided	Incremental QALYs	ICER (€ per BTcP avoided)	ICER (€ per QALY gained)
**Alternative scenario 1: BTcP duration of 15minutes**					
**FST**	-€ 108.43	-3.44%	0.0014	Dominated by FCSL	Dominated by FCSL
**FBSF**	-€ 815.31	-7.51%	0.0027	Dominated by FCSL	Dominated by FCSL
**FBT**	-€ 605.18	-3.69%	0.0014	Dominated by FCSL	Dominated by FCSL
**OTFC**	-€ 579.60	-4.33%	0.0017	Dominated by FCSL	Dominated by FCSL
**Alternative scenario 2: BTcP duration of 45minutes**					
**FST**	-€ 108.43	-5.49%	0.0023	Dominated by FCSL	Dominated by FCSL
**FBSF**	-€ 815.31	-12.03%	0.0044	Dominated by FCSL	Dominated by FCSL
**FBT**	-€ 605.18	-1.57%	0.0008	Dominated by FCSL	Dominated by FCSL
**OTFC**	-€ 579.60	-2.04%	0.0008	Dominated by FCSL	Dominated by FCSL
**Alternative scenario 3 BTcP duration of 60 minutes**					
**FST**	-€ 108.43	-5.82%	0.0023	Dominated by FCSL	Dominated by FCSL
**FBSF**	-€ 815.31	-11.89%	0.0046	Dominated by FCSL	Dominated by FCSL
**FBT**	-€ 605.18	2.68%	-0.0012	€ 22,577.36	€ 500,759.97^
**OTFC**	-€ 579.60	1.26%	-0.0004	€ 46,128.80	€ 1,449,336.12^
**Alternative scenario 4: Health care resource estimated as function of %BTcP reduction**.					
**FST**	€ 50.21	-5.10%	0.0018	€ 983.91	€ 27,611.37
**FBSF**	€ 60.12	-11.29%	0.0039	€ 532.70	€ 15,355.20
**FBT**	-€ 94.08	-4.46%	0.0015	Dominated by FCSL	Dominated by FCSL
**OTFC**	-€ 76.02	-4.82%	0.0018	Dominated by FCSL	Dominated by FCSL
**Alternative scenario 5: time horizon 30 days**					
**FST**	-€ 72.28	-5.10%	0.0012	Dominated by FCSL	Dominated by FCSL
**FBSF**	-€ 543.54	-11.29%	0.0026	Dominated by FCSL	Dominated by FCSL
**FBT**	-€ 403.46	-4.46%	0.0010	Dominated by FCSL	Dominated by FCSL
**OTFC**	-€ 386.40	-4.82%	0.0012	Dominated by FCSL	Dominated by FCSL
**Alternative scenario 6: time horizon 60 days**					
**FST**	-€ 144.57	-5.10%	0.0024	Dominated by FCSL	Dominated by FCSL
**FBSF**	-€ 1,087.07	-11.29%	0.0052	Dominated by FCSL	Dominated by FCSL
**FBT**	-€ 806.91	-4.46	0.0019	Dominated by FCSL	Dominated by FCSL
**OTFC**	-€ 772.80	-4.82%	0.0024	Dominated by FCSL	Dominated by FCSL

FCSL = Sublingual Fentanyl Citrate; BTcP = Breakthrough cancer Pain; QALYs = Quality Adjusted Life Years; FST = Fentanyl Sublingual Tablets; FBSF = Fentanyl Buccal Soluble Film; FBT = Fentanyl Buccal Tablet; OTFC = Oral Transmucosal Fentanyl Citrate; Dominated by FCSL: FCSL was more effective and less costly than the comparator;

^FCSL is less effective and less costly compare to FBT and OTFC, however, compared with FCSL, FBT and OTFC, are not cost-effective because the incremental efficacy is a lot lower than incremental costs.

In the alternative scenarios 5 and 6, FCSL was still the dominant option compared to the others, assuming treatment duration of 30 and 60 days.

### Probabilistic sensitivity analysis

**Fig 2:**
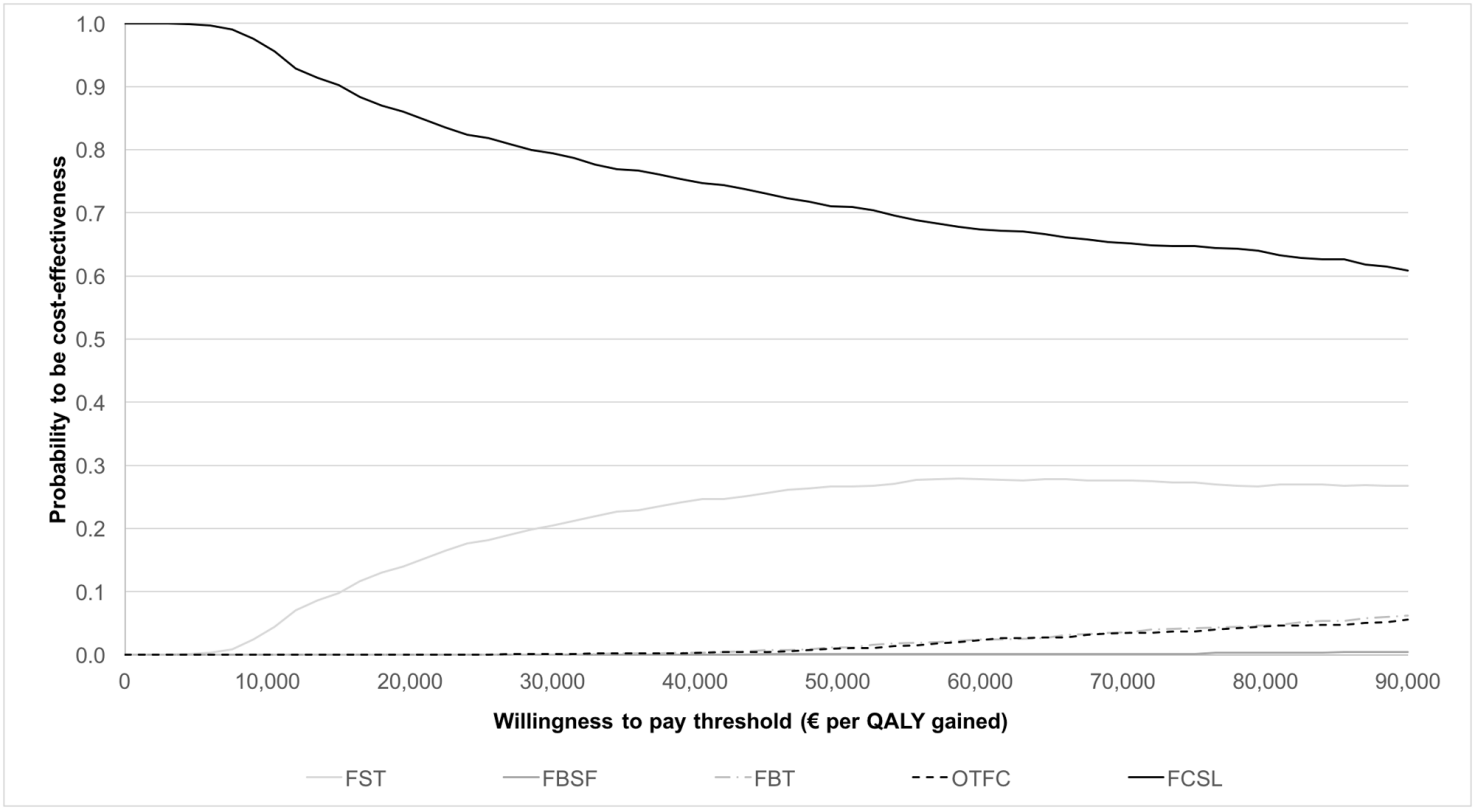
CEAC at base-case scenario. FCSL = Sublingual Fentanyl Citrate; FBSF = Fentanyl Buccal Soluble Film; FBT = Fentanyl Buccal Tablet; OTFC = Oral Transmucosal Fentanyl Citrate; FST = Fentanyl Sublingual Tablets.

The results of probabilistic sensitivity analysis show the impact of the model parameters uncertainty on the results expressed as € per QALY gained (Fig 2). Despite the data uncertainty, the analysis reported FCSL as the therapy with the greater probability of being cost-effective in all willingness to pay threshold assessed (from €0 to €90,000 per QALY gained).

## Discussion

BTcP prevalence is higher than 50% in cancer population, with a high variability of clinical features and a mechanism of onset not always clear [7,31]. In addition, BTcP has a relevant impact on health care costs, a negative impact on quality of life and medical outcomes [8,13,15–19]. Within this scenario, BTcP required the development of a specific approach to its management and an assessment of the treatment options available in order to optimize the health care resources.

To our knowledge this is the first study that assessed the cost-effectiveness of all oral fentanyl formulations available for the treatment of BTcP. In the developed model, the different PI reduction profiles of the fentanyl formulations were linked to the quality of life and costs associated to patients with BTcP in order to identify the most cost-effective formulation. The results reported a lower cost (€1,960.76 per patient) and a higher efficacy (18.7% of BTcP avoided and 0.0507 QALYs) for the FCSL formulation compared to FST, FBSF, FBT and OTFC. The higher efficacy was related to a faster effect in reducing PI, with a lower AUC at 30 minutes compared to other oral formulations (Fig 1) [28,29]. Considering that a recent Italian survey reported an average of 30 minutes for the untreated BTcP episodes [27]; the rapid treatment effect and the PI reduction in the first 30 minutes of a BTcP episode is essential to a better control of such events.

This model is based on a correlation between %BTcP reduction, patient’s Quality of life improvement and costs, with an approach similar to the one used in the only published cost-effectiveness analysis of fentanyl formulations in patients with BTcP [17]. In that study, conducted by Vissers and colleagues, 2 oral formulations (OTFC and FBT) were compared with an Intra-Nasal Fentanyl Spray (INFS) [17]. The most cost-effective option was INFS that was less costly and more effective than OTFC and reported an ICER of €12,203 per gained QALY compared to FBT. Further, FBT resulted as the cost-effective option compared to OTFC because it was both more effective and less costly. Instead, in our study FBT was more effective than OTFC, as reported by Vissers et al. [17], but not less expensive. This difference was due to a different drug price of FBT and OTFC used in the two studies. In our analysis FBT had a higher price than OTFC, while in the Visser et al. study it was the opposite [17]. However, in the Visser et al. study [17] FBT price was based on an assumption because the product was not available in Sweden at the time of the study.

The reliability of the base case results obtained with our model was tested with an extended number of sensitivity analyses (Table 5). In these analyses we assessed different scenarios, changing the treatment duration, the BTcP duration and the health care resources consumed. In all the analyses, FCSL resulted as the cost-effective option compared to the other formulation. The major impact on the results were reported in the scenario with a BTcP duration of 60 minutes and in the scenario with the health care resource estimated as function of %BTcP avoided.

This study had some issues that should be discussed. First, the impact of fentanyl formulation on utility values was derived by Vissers et al. that estimated this data with the TTO method in a UK general population [17]. More reliable data could be collected using a recommended generic quality of life questionnaire as the EQ-5D [38]; however, at the moment of the analysis, the data provided by Vissers et al. [17] were the better available evidences documented with a well-established method [39]. Second, the health care resources included in the model for each fentanyl formulation were derived by the literature and based on the analgesic onset following the approach reported in a previous budget impact analysis [19]. No specific study was performed to estimate the different health care resources consumption within the different fentanyl formulations, even if all the other economic evaluations based their analysis on the relationship between reduction of BTcP analgesic onset and reduction in health care resources consumption [17,18]. To test the possible bias related to the method used to estimate the health care resources consumption in the base-case, we performed a sensitivity analysis estimating the health care consumption based on %BTcP avoided (Table 5), as performed in a previous cost-effectiveness analysis [17]. This sensitivity analysis confirmed the reliability of our base case results, reporting FCSL as the most cost-effectiveness option. A specific study, designed to better estimate this relationship is still needed to improve this type of analyses; however, the method used in our model to assess the other health care resources consumption is the most used and well accepted [15–18]. In addition, adverse events and costs associated with clinical management of adverse effects were not considered in the model, since no relevant data could be identified in the published literature. Most of reported adverse effects are not serious and involved a low cost [18].

Further, no intra-nasal formulations were included in the study. The use of sublingual or oral administration instead of the intra-nasal is related to other aspects than only the lowering pain intensity. Some patients could have contraindication of intra-nasal formulation, other patients can prefer the sublingual or oral administration instead of the intra-nasal and some patients can have a problem in the use of the device for intra-nasal formulation [19,25,40]. These aspects are really difficult to include in a cost-effectiveness model. For this reason, we decided to assess only the sublingual or oral formulation in order to compare rapid onset opioids with a similar way of administration that can be considered similar in terms of preferences and contraindication. At least, we not included immediate-release morphine because it was not recommended for BTcP due to its pharmacokinetic patterns; while it is suggested to be restricted to those cases of predictable, procedure-related pain that persist beyond 60 min [41]. However, there are concerns to the higher cost of fentanyl formulations compare to immediate release morphine [42] that make the use of immediate-release morphine questionable; but we need more suitable clinical data to consider this option in the cost-effectiveness analysis of BTcP in patient with advanced cancer stage.

In conclusion, our study showed the cost-effectiveness of oral fentanyl formulation with a rapid analgesic onset and PI reduction in the first 30 minutes, reporting FCSL as the most cost-effective option, from the Italian NHS point of view. These results can help clinicians and health decision makers to better allocate the resources available to treat these patients. However, more effort should be put in the future researches to understand the economic and epidemiologic impact of BTcP and provide more robust data on the different impact in terms of patients’ quality of life, costs and compliance of the different fentanyl formulations.

## Supporting information

S1 Fig Model structure. (DOCX)

S1 Table Resource use consumed in 45 days using the %BTcP avoided approach. (DOCX)

S2 Table Parameters for probabilistic sensitivity analysis. (DOCX)

S1 File Utility methods. (DOCX)
