# Supplementary material for: Cost-effectiveness analysis of oral fentanyl formulations for breakthrough cancer pain treatment
Source: PLoS One. 2017 Jun 27;12(6):e0179523. doi: 10.1371/journal.pone.0179523 (PMC5487011; doi:10.1371/journal.pone.0179523)
Supplement: S1 Fig — (DOCX) [file pone.0179523.s001.docx]

**S1 Fig.: Model structure**

BTcP= Breakthrough cancer Pain; FCSL=Sublingual Fentanyl Citrate; OTFC=Oral Transmucosal Fentanyl Citrate; FBSF=Fentanyl Buccal Soluble Film; FST=Fentanyl Sublingual Tablets; FBT=Fentanyl Buccal Table.
