# Supplementary material for: Cost-effectiveness analysis of oral fentanyl formulations for breakthrough cancer pain treatment
Source: PLoS One. 2017 Jun 27;12(6):e0179523. doi: 10.1371/journal.pone.0179523 (PMC5487011; doi:10.1371/journal.pone.0179523)
Supplement: S1 Table — (DOCX) [file pone.0179523.s002.docx]

**S1 Table: Resource use consumed in 45 days using the %BTcP avoided approach [17]**

| **Health care Resources, N** | **OTFC** | **FST** | **FBT** | **FBSF** | **FCSL** |
| --- | --- | --- | --- | --- | --- |
| GP visits | 3.87 | 3.88 | 3.86 | 4.16 | 3.65 |
| Specialist visits | 2.01 | 2.02 | 2.00 | 2.16 | 1.90 |
| Hospitalizations | 0.38 | 0.38 | 0.38 | 0.41 | 0.36 |
| Access to ER | 0.86 | 0.86 | 0.86 | 0.93 | 0.81 |
| Physiotherapy | 0.23 | 0.23 | 0.23 | 0.25 | 0.22 |
| Psycotherapy | 1.51 | 1.52 | 1.51 | 1.63 | 1.43 |
| Acupuncture | 0.13 | 0.13 | 0.13 | 0.14 | 0.12 |
| Transcutaneous electrical nerve stimulation | 0.04 | 0.04 | 0.04 | 0.04 | 0.04 |

N=Number; OTFC=Oral Transmucosal Fentanyl Citrate; FST=Fentanyl Sublingual Tablets; FBT=Fentanyl Buccal Tablet; FBSF=Fentanyl Buccal Soluble Film; FCSL=Sublingual Fentanyl Citrate; GP=General Practioner; ER=Emergency Room.
