# Supplementary material for: Cost-effectiveness analysis of oral fentanyl formulations for breakthrough cancer pain treatment
Source: PLoS One. 2017 Jun 27;12(6):e0179523. doi: 10.1371/journal.pone.0179523 (PMC5487011; doi:10.1371/journal.pone.0179523)
Supplement: S2 Table — (DOCX) [file pone.0179523.s003.docx]

**S2 Table: Parameters for probabilistic sensitivity analysis**

| **Model parameters** | **Base-case value** | **Distribution** | **Parameters** |
| --- | --- | --- | --- |
| Utility _FST (30’) | 0.049 | Beta | α = 95.059 β = 1,848.368 |
| Utility_FBSF (30’) | 0.047 | Beta | α = 95.271 β = 1,939.368 |
| Utility_FBT (30’) | 0.049 | Beta | α = 95.023 β = 1,833.226 |
| Utility_OTFC (30’) | 0.049 | Beta | α = 95.059 β = 1,848.058 |
| Utility_FCSL (30’) | 0.051 | Beta | α = 94.875 β = 1,774.977 |
| GP visits (FST), N | 2.18 | LogNormale | Mean=2.175 SD=0.217 |
| Specialist visits (FST), N | 1.13 | LogNormale | Mean=1.130 SD=0.113 |
| Hospitalizations (FST), N | 0.19 | LogNormale | Mean=0.190 SD=0.019 |
| Access to ER (FST), N | 0.44 | LogNormale | Mean=0.435 SD=0.044 |
| Physiotherapy (FST, N | 0.13 | LogNormale | Mean=0.130 SD=0.013 |
| Psycotherapy (FST), N | 0.85 | LogNormale | Mean=0.850 SD=0.085 |
| Acupuncture (FST), N | 0.08 | LogNormale | Mean=0.075 SD=0.008 |
| Transcutaneous electrical nerve stimulation (FST), N | 0.01 | LogNormale | Mean=0.010 SD=0.001 |
| GP visits (FBSF), N | 4.18 | LogNormale | Mean=4.180 SD=0.418 |
| Specialist visits (FBSF), N | 2.17 | LogNormale | Mean=2.170 SD=0.217 |
| Hospitalizations (FBSF), N | 0.37 | LogNormale | Mean=0.365 SD=0.037 |
| Access to ER (FBSF), N | 0.84 | LogNormale | Mean=0.835 SD=0.084 |
| Physiotherapy (FBSF), N | 0.25 | LogNormale | Mean=0.245 SD=0.025 |
| Psycotherapy (FBSF), N | 1.63 | LogNormale | Mean=1.630 SD=0.163 |
| Acupuncture (FBSF), N | 0.14 | LogNormale | Mean=0.140 SD=0.014 |
| Transcutaneous electrical nerve stimulation (FBSF), N | 0.03 | LogNormale | Mean=0.030 SD=0.003 |
| GP visits (FBT), N | 2.98 | LogNormale | Mean=2.975 SD=0.298 |
| Specialist visits (FBT), N | 1.55 | LogNormale | Mean=1.545 SD=0.155 |
| Hospitalizations (FBT), N | 0.26 | LogNormale | Mean=0.260 SD=0.026 |
| Access to ER (FBT), N | 0.60 | LogNormale | Mean=0.595 SD=0.060 |
| Physiotherapy (FBT), N | 0.18 | LogNormale | Mean=0.175 SD=0.018 |
| Psycotherapy (FBT), N | 1.16 | LogNormale | Mean=1.160 SD=0.116 |
| Acupuncture (FBT), N | 0.10 | LogNormale | Mean=0.100 SD=0.010 |
| Transcutaneous electrical nerve stimulation (FBT), N | 0.01 | LogNormale | Mean=0.005 SD=0.001 |
| GP visits (OTFC), N | 2.98 | LogNormale | Mean=2.975 SD=0.298 |
| Specialist visits (OTFC), N | 1.55 | LogNormale | Mean=1.545 SD=0.155 |
| Hospitalizations (OTFC), N | 0.26 | LogNormale | Mean=0.260 SD=0.026 |
| Access to ER (OTFC), N | 0.60 | LogNormale | Mean=0.595 SD=0.060 |
| Physiotherapy (OTFC), N | 0.18 | LogNormale | Mean=0.175 SD=0.018 |
| Psycotherapy (OTFC), N | 1.16 | LogNormale | Mean=1.160 SD=0.116 |
| Acupuncture (OTFC), N | 0.10 | LogNormale | Mean=0.100 SD=0.010 |
| Transcutaneous electrical nerve stimulation (OTFC), N | 0.03 | LogNormale | Mean=0.027 SD=0.003 |
| GP visits (FCSL), N | 1.54 | LogNormale | Mean=1.535 SD=0.154 |
| Specialist visits (FCSL), N | 0.80 | LogNormale | Mean=0.800 SD=0.080 |
| Hospitalizations (FCSL), N | 0.14 | LogNormale | Mean=0.140 SD=0.014 |
| Access to ER (FCSL), N | 0.31 | LogNormale | Mean=0.310 SD=0.031 |
| Physiotherapy (FCSL), N | 0.09 | LogNormale | Mean=0.090 SD=0.009 |
| Psycotherapy (FCSL), N | 0.60 | LogNormale | Mean=0.600 SD=0.060 |
| Acupuncture (FCSL), N | 0.05 | LogNormale | Mean=0.050 SD=0.005 |
| Transcutaneous electrical nerve stimulation (FCSL), N | 0.01 | LogNormale | Mean=0.010 SD=0.001 |

N= Number; FST=Fentanyl Sublingual Tablets; FBSF=Fentanyl Buccal Soluble Film; FBT=Fentanyl Buccal Tablet; OTFC=Oral Transmucosal Fentanyl Citrate; FCSL=Sublingual Fentanyl Citrate; GP=General Practioner; ER=Emergency Room.
