# Supplementary material for: Cost-effectiveness analysis of oral fentanyl formulations for breakthrough cancer pain treatment
Source: PLoS One. 2017 Jun 27;12(6):e0179523. doi: 10.1371/journal.pone.0179523 (PMC5487011; doi:10.1371/journal.pone.0179523)
Supplement: S1 File — (DOCX) [file pone.0179523.s004.docx]

**S1 File: Utility methods**

Based on Visser et al study [17], we estimated utility values associated to the different %BTcP avoided. Visser et al. reported the QALYs obtained in 180 days and the %BTcP avoided associated to that QALYs gained (see the following Table).

Because outcome discount rate and mortality probability was not applied in the Visser et al. simulation, the QALYs estimated represented the 50% of the utility value gain with the associated BTcP reduction (considering the time horizon of 180 days using in the base case of the Visser et al. simulation).

Based on these data and on the %BTcP avoided and QALYs association reported in the treatments analyzed by Visser et al., we built a graph to extrapolate the function that put in relation utility and %BTcP avoided. We multiply the extrapolated function by 2 to taking into account that utility reported by Visser et al. was associated to a time horizon of half year.

**QALY and %BTcP avoided reported in the Visser et al. study [17]**

|  | **INFS** | **OTFC** | **FBT** |
| --- | --- | --- | --- |
| **% BTcP avoided vs Placebo (95 % UI)** | 55.00 (46.00, 68.00)% | 29.00 (22.00, 38.00)% | 31.00 (25.00, 39.00)% |
| **QALYs** | 0.266 (0.251, 0.281) | 0.220 (0.203, 0.253) | 0.223 (0.209, 0.237) |

INFS= Intra-Nasal Fentanyl Spray; OTFC=Oral Transmucosal Fentanyl Citrate; FBT=Fentanyl Buccal Tablet; BTcP=Breakthrough cancer Pain; UI= 95% Uncertainty Intervals; QALYs=Quality Adjusted Life Years.


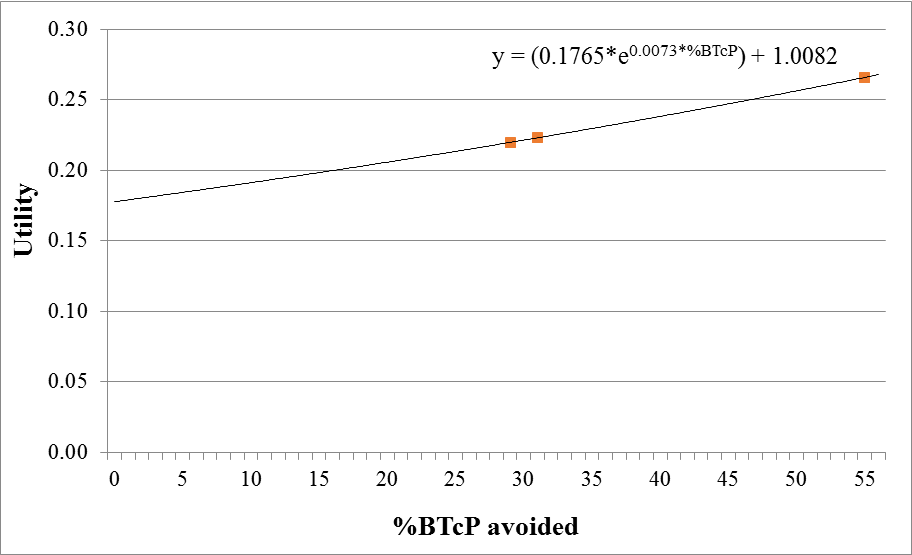


**Graph used to estimate the utility function**

y=utility, BTcP= Breakthrough cancer Pain; %BTcP=percentage of BTcP avoided.
